# Rapid improvement of psychiatric stigmata after IFN-free treatment in HCV patients with and without cryoglobulinemic vasculitis

**DOI:** 10.1007/s10067-021-05877-3

**Published:** 2021-08-19

**Authors:** Laura Gragnani, Serena Lorini, Lorenzo Martini, Cristina Stasi, Marcella Visentini, Luisa Petraccia, Niccolò Marello, Monica Monti, Silvia Marri, Francesco Madia, Valdo Ricca, Anna Linda Zignego

**Affiliations:** 1 MASVE Interdepartmental Hepatology Center, Department of Experimental and Clinical Medicine, University of Florence, Center for Research and Innovation CRIA-MASVE, Largo Brambilla 3, 50134 Firenze, Italy; 2 Department of Translational and Precision Medicine, Sapienza University of Rome, 00185 Rome, Italy; 3 Department of Health Sciences, Psychiatry Unit, University of Florence, 50134 Florence, Italy

**Keywords:** Cryoglobulinemia, Depression, Liver disease, Quality of life, Vasculitis

## Abstract

**Objective:**

Hepatitis C virus (HCV) causes neuropsychiatric disorders and quality of life impairment, especially in patients with cryoglobulinemic vasculitis (CV). Direct acting antivirals (DAAs) are effective in most extrahepatic HCV diseases, but limited information exists regarding the outcome of psychiatric disorders in patients with and without CV, after therapy. We aimed to evaluate psychiatric outcomes, in HCV-patients with and without CV, before and after successful DAA therapy.

**Methods:**

We prospectively studied DAA-treated HCV-patients, stratified into presence (CV) or absence of CV (NON-CV). Four psychometric scales were administered to assess depression (HAM-D and MADRS), anxiety (HAM-A), and mania (MRS). Short-Form-36 questionnaires evaluated quality of life.

**Results:**

Seventy-six patients were recruited, and 47 CV and 29 NON-CV were treated with antivirals. At baseline, depression and anxiety, from mild to severe, were frequently shown, with the most advanced cases in thee CV group; no patients achieved the scores for mania. A significant improvement emerged for all the psychometric scales in the entire population and in the subgroups, after viral eradication even in the short-term outcome. The Short-Form-36 summary components showed benefits.

**Conclusions:**

After HCV eradication, the depression and anxiety scores significantly improved and severity grade generally lowered. DAA-positive effects on mental disorders should be considered part of the therapy outcome, being beneficial especially in CV patients who usually have worse baseline mental scores.

**Table:** 

**Key Points** • HCV frequently causes psychiatric disorders and an often-invalidating autoimmune/lymphoproliferative disease called cryoglobulinemic vasculitis. • The new direct acting antivirals (DAAs) are very effective and well tolerated by HCV-patients. • This study shows DAA-induced benefits on depression and anxiety in HCV-patients that are especially evident in CV patients who usually have worse baseline mental scores. • DAA-induced benefits are observed in the short-term post-therapy follow-up, in contrast with data previously obtained in HCV patients treated with IFN-based anti-HCV therapy.

**Supplementary Information:**

The online version contains supplementary material available at 10.1007/s10067-021-05877-3.

## Introduction

Hepatitis C virus (HCV) infection is considered a systemic disease due to the involvement of other organs and tissues concomitantly with liver disease [1, 2]. Among the extrahepatic manifestations, neuropsychiatric disorders have been reported in up to 50% of chronic HCV-infected patients in different studies [3]. The prevalence of HCV infection in psychiatric populations ranges from 6.7 to 8.5%, which is threefold higher than in the general population [4].

The most frequent neuropsychiatric disorders are cognitive impairment (brain fog), depression, anxiety, and fatigue, which mainly manifest as physical and mental exhaustion and are often in association with attention deficit and sleep disturbances (60% of cases) [5, 6].

Even if the brain is a suitable site for HCV replication and the virus may directly exert neurotoxicity [7], other mechanisms have been proposed to explain the pathogenesis of HCV-related neuropsychiatric involvement [8]. The latter includes derangement of metabolic pathways of infected cells, alterations in neurotransmitter circuits, autoimmune disorders, and mostly cerebral or systemic inflammation through the action of cytokines [9]. In fact, significant correlations have been reported between high choline/creatine ratio in white matter and HCV replication; high choline/creatine ratio was also associated with higher degrees of cognitive dysfunction in HCV patients and may be implicated in glial activation secondary to oxidative stress [9]. Furthermore, N-acetyl aspartate (NAA), a marker of functional integrity of nervous cells and pathways, and its low levels are associated with memory deficiency [9]. In chronic HCV-infected patients, a significantly decreased NAA/creatine ratio in the cerebral cortex correlated with cognitive impairment, anxiety, and depression, with respect to healthy controls [9].

In addition, increased choline and myo-inositol levels in basal ganglia and white matter as well as altered concentrations of creatine and NAA in basal ganglia, indicative of glial activation and macrophage infiltration, were found in patients with HCV-chronic infection and neuropsychiatric disorders [9].

Of course, chronic HCV infection triggers a systemic and local inflammation resulting in the production of such cytokines like IL-1, IL-6, IL-4, and TNF-α, which are responsible for the neuronal changes underlying neurological impairment [10] since peripheral proinflammatory cytokines, like IL-1 and IL-6, can interfere with neurotransmitter systems thus predisposing to neuropsychiatric disorders [9, 10].

The most frequent HCV-related extrahepatic manifestation is cryoglobulinemic vasculitis (CV), which is the systemic inflammation of the small/medium vessels due to the deposit of immune complexes called cryoglobulins and complement factors on the vascular endothelium [2]. Although the correlation between HCV infection and CV is very strong, HBV-related (in about 5% of cases) [11] as well as idiopathic CV also exists [12].

Different symptoms arise from the involvement of various organs and systems, namely the skin, joints, kidneys, the nervous system, and the salivary/lachrymal glands. Among CV symptoms, central nervous system vasculitis is rare and presents variable neurological manifestations depending on size and location of involved vessels within the nervous system, which makes the diagnosis quite challenging [13].

Hence, the symptoms defining full-blown CV can be so numerous and severe as to determine an extremely poor quality of life for the patient [14].

In general, HCV has been shown to have a relevant impact on patient‐reported outcomes, such as health‐related quality of life (HQoL) and fatigue [15]. The role of Direct-Acting Antivirals (DAAs) in lowering this negative impact can be appreciated by combining clinical outcomes and PROs [16] and the effect of treatment should be considered as part of the therapeutic outcome [17].

Although, among HCV patients, those with CV have lower pre-therapy HQoL scores concerning both physical and mental component summaries [14, 18], the HQoL improvement is significantly evident in the CV setting after a DAA-induced sustained virological response (SVR) [18].

Therefore, DAA-based therapy is well tolerated and effective on different aspects of the HCV-disease even in hard to manage patients with severe extrahepatic manifestations [18–21], overcoming the well-known side effects of interferon (IFN)-based treatment. In fact, among others, depression and other psychiatric symptoms were frequent side effects of IFN therapy and often affected eligibility to therapy and patients’ compliance [22].

At present, there are no studies that describe, through the administration of diagnostic psychiatric self-reported scales, the effects of successful DAA treatment on psychiatric disorders in different subgroups of HCV-chronically infected patients. Therefore, the aim of this study was to analyze the behavior of four psychiatric scales in HCV-patients successfully undergoing DAA therapy and stratified on the basis of the presence/absence of cryoglobulinemic vasculitis, since the CV patients previously showed lower HQoL levels also regarding the mental component summary.

## Patients and methods

Between January 2018 and March 2019, we enrolled consecutive patients with detectable levels of serum HCV‐RNA and eligibility for IFN‐free antiviral treatment in the outpatient clinic at the Interdepartmental Center for Systemic Manifestations of Hepatitis Viruses (MaSVE), University of Florence, Florence, Italy. The patients were evaluated at four different time points: before the initiation of treatment week 0 (W0), at week 4 of treatment (W4) at the end of therapy (EOT), and at week 12 of the post-therapy follow-up when the SVR was established (SVR12). A complete blood test was performed to assess liver- and CV-related main parameters concomitantly with routine check-ups.

The CV clinical response was defined as previously described [18, 23]. Briefly, complete clinical response (CR) was assessed when all baseline clinical manifestations were improved, with the distinction of a full complete clinical response (FCR) when all the symptoms disappeared (“*restitution ad integrum*”), a partial clinical response (PR) with an improvement in at least half of the baseline symptoms, and no clinical response (NR) in the remaining cases.

The patients were interviewed at the same time points in order to complete the psychometric scales and to assess HQoL through specific questionnaires. No patients received anxiolytics/antidepressants during the DAA therapy and during the period of follow-up described in this study. Three patients who initiated treatment for depression after DAAs, following a psychiatric evaluation, were excluded from the analysis since questionnaire results could have been altered by the drugs.

The study was conducted according to the 1975 Declaration of Helsinki; all treatments were open‐label, provided by the National Health Care System, and did not require ethical approval. The study received institutional review board approval (evaluation code: SPE 14.084_ AOUC); informed consent was obtained from all patients.

### Psychometric assessment

Several reliable and validated psychometric scales are widely used tools in both clinical and research settings. In this study, four different psychometric scales were administered to assess the presence and the severity of psychiatric symptoms as well as to measure the therapeutic outcome.

#### Hamilton Depression Rating Scale

The Hamilton Depression Rating Scale (Ham-D) is the most widely used clinician-administered depression assessment scale. The original version contains 17 items pertaining to symptoms of depression experienced over the past week [24, 25], but modified versions are available differing in number, sequence, wording of items, and in the scoring procedure [25]. We used the HAM-D version in which 21 items are included [26], although the extra 4 symptoms (i.e., diurnal variation, depersonalisation/derealisation, paranoid, and obsessional/compulsive symptoms) are not included in the score calculation as they were not considered features related to depression severity [27]. The cut-off scores (adjusted on the basis of the judgment of an experienced clinician) reflect different levels of depression severity as follows: 0–7 = normal; 8–16 = mild depression;17–23 = moderate depression; ≥ 24 = severe depression [28].

#### Hamilton Anxiety Rating Scale

Developed in 1959 by Dr. M. Hamilton [29], Hamilton Anxiety Rating Scale (HAM-A) is a widely used and well-validated tool for measuring the severity of a patient’s anxiety. The main benefit of HAM-A is to assess a patient's response to treatment, and by administering the scale serially, it is possible to document the results of drug treatment or psychotherapy [30]. The scale consists of 14 items, each defined by a series of symptoms, and each item is scored on a 5-point scale, ranging from 0 = not present to 4 = severe [30]. The optimal HAM-A score ranges were: mild anxiety = 8–14; moderate = 15–23; and severe ≥ 24 (scores ≤ 7 were considered to represent no/minimal anxiety).

#### Montgomery-Asberg Depression Rating Scale

Montgomery-Asberg Depression Rating Scale (MADRS) is suitable for the evaluation of adult patients with symptoms of depression. It was developed through a collaboration among English and Swedish schools [31] and it consists of nine questions, each scored from 0 to 6, whereby a higher score indicates more severe symptoms. The total MADRS-S score, according to the developers, should be interpreted as follows: 0–6 no depression, 7–19 mild depression, 20–34 moderate depression, and > 34 severe depression [31].

#### Mania Rating Scale

The Bech–Rafaelsen Mania Rating Scale (MRS) is the most commonly used scale for assessing treatment response concerning mania symptoms [32]. It was developed in 1979 to provide a clinical severity rating and it consists of 11 items rated on a five-point scale with “0” indicating normal mood and behavior and “4” indicating severe impairment [32]. The total score reflects the severity of mania as either mild [15, 16], moderate [17–20, 22, 24, 25], marked [26–29], severe [30–39], or extreme (≥ 44) [32, 33].

#### Measurements and analysis of HQoL

Patients were interviewed with a patient‐reported outcome questionnaire at the four time-points as previously described [18]. We adopted a widely used generic/validated instrument for HQoL evaluation, the Short Form‐36 version 2 (SF‐36v2) [34]. It assesses 8 HQoL scales (scores from 0 to 100 with higher values corresponding to better health status): physical functioning, role physical, bodily pain, general health, vitality, social functioning, role emotional, and mental health. Two summarizing scores are derived from the eight subscales: a physical health component score (PCS) and a mental health component score (MCS) [35]. In general, higher scores on all subscales represent better health and functioning.

#### Statistical analysis

Quantitative variables were analyzed using one‐way analysis of variance (nonparametric ANOVA or Friedman test) or with t- or Wilcoxon test for unpaired samples. Categorical variables were analyzed with the *χ*^2^ test and Fisher’s exact test when necessary. All tests were two‐sided at a 0.05 significance level. Analyses were performed by Stata v.9.0 (StataCorpLP, College Station, TX, USA). The Pearson’s correlation test was used to measure the strength of the association between two variables. Scores were obtained from the SF‐36v2 questionnaire from dedicated software and then statistical analysis was performed.

## Results

Seventy-six (76) consecutive HCV-positive patients needing DAA-based anti-HCV therapy were enrolled between January 2018 and March 2019 at the outpatient clinic of the Interdepartmental Center for Systemic Manifestations of Hepatitis Viruses (MaSVE) at the University of Florence and Hepatology Research and Innovation Center at Careggi Hospital, Florence, Italy. A flow diagram describing the enrollment details and evaluation time-points is provided as Online Resource.

All the 76 HCV patients obtained a SVR, which is defined as undetectable viremia 12 weeks after the EOT.

The main socio-demographic, hepato-virological, and clinical characteristics of the population are described in Table 1. No patients had concomitant co-infections.

**Table 1:** Main demographic, hepatovirological, and clinical baseline characteristics of the total HCV population and the groups stratified based on the presence/absence of CV

		Total HCV population *n* = 76	HCV-CV *n* = 47	HCV NON-CV *n* = 29	*p* CV vs NON-CV
Mean age (years)		65.1 (± 1.5)	70.28 (± 1.58)	63.41(± 2.70)	
Gender (M/F)		47 (62%)/29 (38%)	17(36%)/30(64%)	11(38%)/18(62%)	
Metavir^	F0-F1	25 (32.9%)	20 (42%)	6 (21%)	
	F2	13 (17.1%)	5 (11%)	7 (24%)	
	F3	18 (23.7%)	9 (19%)	9 (31%)	
	F4	20 (26.3%)	13 (28%)	7 (24%)	
ALT^§^ (U/L)		126.8 (± 15.0)	68.53 (± 7.223)	105.2 (± 21.45)	
AST^£^(U/L)		87.8 (± 7.6)	55.87 (± 4.98)	81.56 (± 13,88)	
Total bilirubin^°^(mg/dL)		1.13 (± 0.2)	1.26 (± 0.30)	0.89 (± 0,07)	
Direct bilirubin^$^ (mg/dL)		0.27 (± 0.02)	0.26 (± 0.03)	0.27 (± 0,04)	
HCV-RNA (U/mL × 10^6^)		3.41 (± 0.44)	2.58 (± 0.92)	5.06 (± 0,44)	*
HCV genotype					
	1a	15 (19.8%)	7 (15%)	7 (24%)	
	1b	41 (53.9%)	23 (49%)	17 (59%)	
	2	14 (18.5%)	12 (26%)	2 (7%)	
	3	4 (5.2%)	3 (6%)	2 (7%)	
	4	2 (2.6%)	2 (4%)	1 (3%)	
Co-therapy					
Glucocorticoids		10 (13%)	10 (21%)	-	***
Antihypertensive drugs		12 (16%)	7 (15%)	5 (17%)	
Hypoglycemic drugs		8 (11%)	5 (11%)	3 (10%)	
Previous intravenous drug use		12 (16%)	5 (11%)	7 (24%)	
Previous IFN-based treatment		29 (38%)	17 (36%)	12 (41%)	
Previous IFN-related psychiatric anamnesis^#^		9 (29%)	6 (13%)	3 (10%)	
Cryocrit		-	3% (± 0.82)	-	**
Rheumatoid factor		-	255.1(± 140.1)	< 20	***
C4 consumption^		-	13 (28%)	5 (17%)	

Data are expressed as number, percentage and, when required, mean and standard error of mean. ^ Based on liver stiffness assessed by FibroScan; ^**§**^*ALT* alanine aminotransferase, normal range: 12–65 U/L; ^£^*AST* aspartate aminotransferase, normal range: 15–37 U/L; ^**§**^ Total bilirubin normal range: 0.2–1 mg/dL; ^**§§**^ Direct Bilirubin normal range: 0–0.2 mg/dL; IFN: Interferon; ^#^ Calculated on the 29 patients previously treated with IFN; ^ number of patients with C4 value under 0.10 g/L (normal range: 0.10–0.40 g/L). ^*^ *p* = 0.007; ** *p* = 0.0001; *** *p* = 0.0008

### Total HCV population analysis

**Fig. 1:**
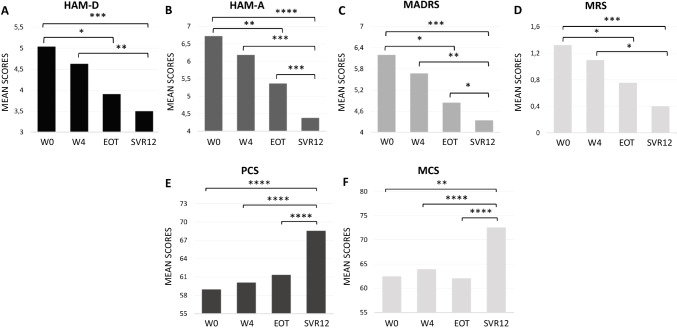
Average scores of the psychometric scales and of the synthetic indexes of the Short Form-36 questionnaire, PCS and MCS, for the entire population over time. Panel **A** HAM-D mean values; panel **B** HAM-A mean values; panel **C** MADRS mean values; panel **D** MRS mean values; panel **E** PCS mean scores; panel **F** MCS mean scores; ^*^ = *p* ≤ 0.05, ^**^ = *p* ≤ 0.01, ^***^ = *p* ≤ 0.001, **** = *p* ≤ 0.0001. Abbreviations: HAM-D (Hamilton Rating Scale for Depression), HAM-A (Hamilton Rating Scale for Anxiety); MADRS (Montgomery-Asberg Depression Rating Scale); MRS (Mania Rating Scale); PCS (Physical Component Summary); MCS (Mental Component Summary; W0 (Week 0); W4 (Week 4); EOT (End of Treatment); SVR12 (Sustained Virological Response 12 weeks)

The analysis of the psychometric scales on the total population showed a significant improvement of scores in all administered standards (Fig. 1, panels A–D), between W0, SVR12, and other time-point comparisons as showed in Fig. 1. The degrees of psychiatric severity at baseline (W0) and at SVR12 are reported in Table 2. At W0, the HAM-D scale scores analysis revealed mild to severe depression in 29% of HCV subjects, the HAM-A derived scores showed mild to severe anxiety in 42% of patients, and the MADRS scales attributed a mild to moderate grade of depression severity in about 37% of patients. None of the patients achieved the score defining the presence of mania symptoms from the MRS analysis (Table 2).

**Table 2:** Severity of depression, anxiety, and mania, resulting from the four psychometric scales, in the general population and in the groups stratified based on the presence/absence of CV

	Total population = 76		CV group *n* = 47		NON-CV group *n* = 29	
	W0	SVR12	W0	SVR12	W0	SVR12
HAM-D						
No depression	54 (71.1%)	62 (81.6%)	34 (72.3%)	36 (76.6%)	20 (69%)	26 (89.7%)
Mild depression	19 (25%)	13 (17.1%)	10 (21.3%)	10 (21.3%)	9 (31%)	3 (10.3%)
Moderate	2 (2.6%)	1 (1.3%)	2 (4.3%)	1 (2.1%)	-	-
Severe	1 (1.3%)	-	1 (2.1%)	-	-	-
HAM-A						
No anxiety	44 (57.9%)	58 (76.3%)	26 (55.3%)	34 (72.3%)	18 (62%)	24 (82.8%)
Mild anxiety	25 (32.9%)	13 (17.1%)	15 (31.9%)	10 (21.3%)	10 (34.5%)	3 (10.3%)
Moderate anxiety	6 (7.9%)	5 (6.6%)	5 (10.6%)	3 (6.4%)	1 (3.5%)	2 (6.9%)
Severe anxiety	1 (1.3%)	-	1 (2.1%)	-	-	-
MADRS						
No depression	48 (63.1%)	59 (77.6%)	29 (61.7%)	35 (74.5%)	19 (65.5%)	24 (82.8%)
Mild depression	26 (34.2%)	15 (19.7%)	16 (34%)	10 (21.3%)	10 (34.5%)	5 (17.2%)
Moderate	2 (2.6%)	2 (2.6%)	2 (4.3%)	2 (4.2%)	-	-
Severe	-	-	-	-	-	-
MRS						
No mania	76 (100%)	76 (100%)	47 (100%)	47 (100%)	29 (100%)	29 (100%)
Mild mania	-	-	-	-	-	-
Moderate mania	-	-	-	-	-	-
Marked mania	-	-	-	-	-	-
Severe mania	-	-	-	-	-	-

Data are expressed as numbers and percentages. Abbreviations: *CV*, cryoglobulinemic vasculitis; *W0*, week 0; *W4*, week 4; *EOT*, end of treatment; *SVR12*, sustained virological response 12 weeks; *HAM-D*, Hamilton Rating Scale for Depression; *HAM-A*, Hamilton Rating Scale for Anxiety; *MADRS*, Montgomery-Asberg Depression Rating Scale; *MRS*, Mania Rating Scale

The number of patients with no symptoms increased from W0 and SVR12 for the two scales to assess depression (HAM-D and MADRS) and for the HAM-A (Table 2).

The SF-36 questionnaire analysis on the total sample is reported as synthetic SF-36 indexes, PCS and MCS, in Fig. 1 panels E and F, respectively. Both PCS and MCS showed an improvement from W0 to SVR12; however, while PCS progressively increases, MCS displayed worsening at the EOT.

### IFN-experienced Vs naïve patient analysis

We divided the total HCV population into two subgroups based on the previous experience of an IFN-based therapy: 29 patients failed an IFN-treatment (HCV-IFN) and 47 were naïve (HCV-naïve). Among the HCV-IFN subgroup, we observed a significant improvement between W0 and SVR12 for the HAM-A and MRS (*p* = 0.03 and *p* = 0.01, respectively). No significant differences were observed regarding the two scales to assess depression (HAM-D and MADRS) although the mean scores showed an improvement. Concerning the HCV-naïve subgroup, we observed a significant improvement between W0 and SVR12 for all the scales (HAM-D *p* = 0.0009, HAM-A *p* = 0.0004, MADRS *p* = 0.001, MRS *p* = 0.003).

The mean score comparison between HCV-IFN and HCV-naïve subgroups, for each time-points, did not show statistically significant differences except for the MRS scale at EOT (*p* = 0.02).

### CV Vs. NON-CV analysis

The sample was divided into two subgroups based on the presence (47 CV patients) or absence of HCV-related mixed cryoglobulinemic vasculitis (29 NON-CV patients). CV diagnosis was assessed as previously described [40]. The two subgroups did not show statistically significant differences for the main demographic and clinical features, except, as expected, for the parameters attributable to CV presence and for HCV viremia as reported in Table 1.

Concerning the clinical response of the CV patients: at the EOT, 7 (15%) were FCR, 9 (19%) were CR, 13 (28%) were PR and partial responders, 18 (38%) were NR; at the SVR12, 19 (40%) were FCR, 14 (30%) were CR, 2 (4%) were PR, and 12 (26%) were NR.

**Fig. 2:**
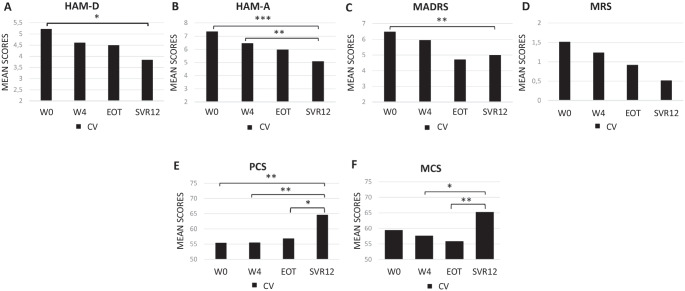
Average scores of the psychometric scale and of the synthetic indexes of the Short Form-36 questionnaire, PCS and MCS, for CV subgroup over time. Panel **A** HAM-D mean values; panel **B** HAM-A mean values; panel **C** MADRS mean values; panel **D** MRS mean values; panel *E* PCS mean scores; panel **F** MCS mean scores; ^*^ = *p* ≤ 0.05, ** = *p* ≤ 0.01, *** = *p* ≤ 0.001. Abbreviations: CV, cryoglobulinemic vasculitis; HAM-D, Hamilton Rating Scale for Depression; HAM-A, Hamilton Rating Scale for Anxiety; MADRS, Montgomery-Asberg Depression Rating Scale; MRS, Mania Rating Scale; PCS, Physical Component Summary; MCS, Mental Component Summary; W0, week 0; W4, week 4; EOT, end of treatment; SVR12, sustained virological response 12 weeks)

**Fig. 3:**
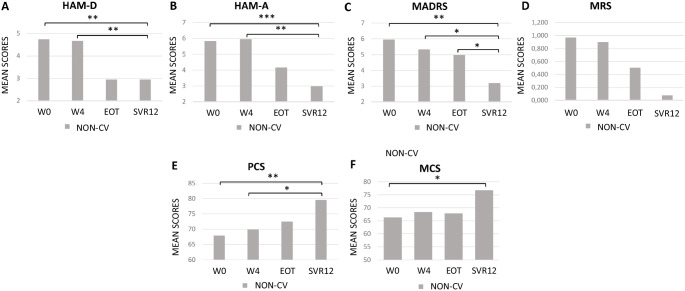
Average scores of the psychometric scale and of the synthetic indexes of the Short **Form-36 questionnaire, PCS and MCS, for NON-CV subgroup over time.** Panel **A** HAM-D mean values; panel **B** HAM-A mean values; panel **C** MADRS mean values; panel **D** MRS mean values; panel **E** PCS mean scores; panel **F** MCS mean scores; * = *p* ≤ 0.05, ** = *p* ≤ 0.01, *** = *p* ≤ 0.001. Abbreviations: NON-CV, non-cryoglobulinemic vasculitis; HAM-D, Hamilton Rating Scale for Depression; HAM-A, Hamilton Rating Scale for Anxiety; MADRS, Montgomery-Asberg Depression Rating Scale; MRS, Mania Rating Scale; PCS, Physical Component Summary; MCS, Mental Component Summary; W0, week 0; W4, week 4; EOT, end of treatment; SVR12, sustained virological response 12 weeks

Considering the behavior of the scores for each subgroup individually, a significant improvement from W0 and SVR12 was observed for the two scales to assess depression (HAM-D and MADRS) and for the HAM-A (Fig. 2 and Fig. 3, panels A–C). No significant differences were observed regarding MRS between W0 and SVR12 (Fig. 2 and Fig. 3, panel D). Statistical significant results of other time-point comparisons are showed in Fig. 2 for the CV subgroup and in Fig. 3 for the NON-CV subgroup.

The severity of psychiatric symptoms, emerging from the scores from the four scales, is reported in Table 2. Among the CV patients, one case of severe depression became moderate and one with severe anxiety improved to moderate (Table 2).

**Fig. 4:**
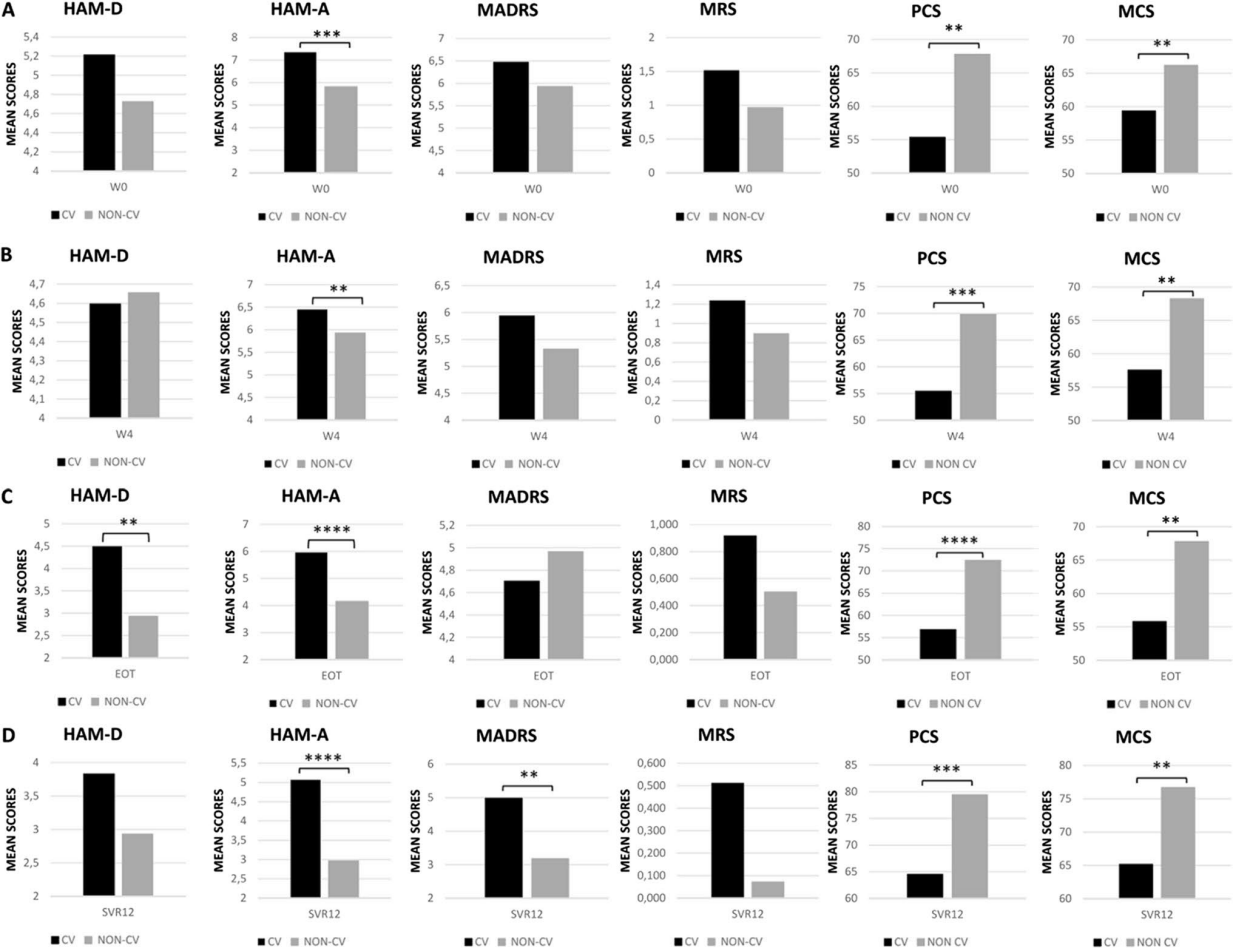
Comparison of the mean scores of the psychometric scales and of the synthetic indexes of the Short Form-36 questionnaire, PCS and MCS, between the CV and NON-CV subgroups for each time-points. CV vs NON-CV mean score resulting from the four psychometric scales and from the summary scores of SF-36; row W0: week 0 analysis; row B W4: week 4 analysis; row EOT: End Of Treatment analysis; row SVR12: Sustained Virological Response 12 weeks analysis. ** = *p* ≤ 0.01, *** = *p* ≤ 0.001, **** = *p* ≤ 0.0001. Abbreviations: CV, cryoglobulinemic vasculitis; NON-CV, non-cryoglobulinemic vasculitis; SF-36, Short Form-36 questionnaire; HAM-D, Hamilton Rating Scale for Depression; HAM-A, Hamilton Rating Scale for Anxiety; MADRS, Montgomery-Asberg Depression Rating Scale; MRS, Mania Rating Scale; PCS, Physical Component Summary; MCS, Mental Component Summary

The results of the SF-36 comparisons between CV and NON-CV mean scores, at each time- points showed that the CV group summarizing scores, PCS and MCS, were lower compared to the NON-CV group, corresponding to a worse quality of life (Fig. 4).

The analysis of the SF-36 questionnaire on the CV and NON-CV subgroups highlighted a significant improvement in PCS and MCS over time (Fig. 2 and Fig. 3, panels E, F).

## Discussion

The prospective analysis we reported is, to the best of our knowledge, the first one regarding the effects of the DAA IFN-free therapy on psychiatric disorders, assessed through a comprehensive panel of validated scales, implemented with HQoL Sf-36 questionnaires, in HCV-patients without a previous psychiatric diagnosis and stratified into presence/absence of CV.

It is well known that the previous standard of care therapy based on IFN often induced psychiatric side effects, resulting in treatment discontinuation and rendering subjects with mental health disorders unsuitable for this regimen except for isolated cases [4]. In fact, IFN possibly exerts a direct central effect like other pro-inflammatory cytokines, determining depression-specific and neurovegetative syndrome [36]. Despite IFN limitations in the setting of psychiatric disorders or psychiatric risk, a few authors reported some benefits caused by an IFN-induced SVR on these extrahepatic manifestations [37, 38]. IFN-based benefits were mostly evident in the long-term follow-up as, at the SVR, pre-treatment HQoL scores were restored and not improved [41].

The introduction of DAA IFN-free regimens broadened eligibility for anti-HCV therapy permitting the treatment of difficult-to-treat subjects such as those with severe CV [39]. DAAs can be administered to patients suffering from mental health illnesses and mood disorders without the risk of psychiatric decompensation [42, 43], although Miarons and colleagues reported mild neuropsychiatric symptoms that do not affect treatment adherence [44].

The results of our prospective analysis showed, from baseline to SVR12, an overall significant improvement in the mean scores of the four psychometric scales that is highly significant for those measuring depression and anxiety; no worsening was recorded from baseline to SVR12. A similar analysis was performed by Sundberg et al. who administered the MADRS and the Pittsburgh Sleep Quality Index, implemented with tests for Alcohol Use and Drug Use Disorders Identification, in 16 DAA-treated HCV patients with a previous history of mental health illness or substance abuse/dependence, and showed a significant reduction of depression at SVR12 [43]. A Patient Health Questionnaire-9 to assess the degree of depression severity was used by Sackey and colleagues to perform an analysis on 48 HCV patients undergoing DAA-therapy, among whom 24 had already received a psychiatric diagnosis [42]. The evaluation was made comparing baseline to the EOT scores and although an improvement was seen in both the patient subgroups (those with and without a history of previous mental illness), the change was not significant [42]. The present study clearly showed the benefits of DAA-induced SVR by analyzing a wider population, in a longer follow-up period than previous studies, and by using four different psychometric scales on patients without a previous mental illness diagnosis (with the exception of some patients experiencing psychiatric side effects from previous IFN-based therapy). Although the improvement of PCS and MCS after DAA-related SVR is not an original observation, the concomitant analysis of HRQoL confirms previous results [15] and strengthens the data obtained from the psychometric scales, since the positive changes on physical wellbeing and mood are in fact perceived by the patients. In the study sample, about 30% of patients suffered from mild to moderate depression at baseline and about 40% had mild to severe anxiety although previously undiagnosed; no patients had symptoms of mania. These results confirm the high frequency of mood and psychiatric disorders in the HCV-infected subjects [45], but they also suggest the need to administer (or self-administer) psychometric scales to HCV patients in order to ensure a complete multidisciplinary management of the so-called HCV disease. In addition, the administration of psychometric scales could be the best way to measure the improvement of psychiatric symptoms over time, especially after therapy.

We previously reported that among HCV patients, those with CV had worse baseline HQoL and interferon‐free therapy was effective in significantly increasing PCS and MCS [18]. The HQoL analysis reported in the present study completely confirmed all the previous results, further stressing the important effects of DAA- induced SVR in improving CV's individual and social burden.

Presently, there are no reports assessing the severity of depression, anxiety, and mania in HCV patients affected by CV nor are there any studies describing the effect of viral eradication of these psychiatric disorders in the CV setting compared to HCV subjects without CV. Higher mean scores for the two scales assessing depression (HAM-D and MADRS) and for the one assessing mania were observed in CV compared to NON-CV patients, even if the difference (possibly due to the population’s size) did not achieve statistical significance. A significantly higher mean score for anxiety (HAM-A) was recorded in the CV group compared to the NON-CV group at all the evaluated time points. Interestingly, in the assessment of mental illness severity, only two patients with severe grade disease (one with depression and one with anxiety) were in the CV group.

In general, a significant improvement of all the scores was evident in both settings, with and without CV, demonstrating an improvement in psychiatric disorder severity as another positive outcome of viral eradication, especially in patients suffering from CV, which is an invalidating condition in many instances. This appears to be of special interest not only for the obvious positive effects on the patients, but also in light of the consistent indirect costs, due to the loss of productivity, correlated to chronic HCV infection, in particular in the setting of CV patients characterized by a lower HQoL and sometimes invalidating clinical manifestations [46].

## Supplementary Information

Below is the link to the electronic supplementary material.

Supplementary file1 (PDF 240 KB)

## Data Availability

Not applicable.
