# Supplementary material for: Rapid improvement of psychiatric stigmata after IFN-free treatment in HCV patients with and without cryoglobulinemic vasculitis
Source: Clin Rheumatol. 2021 Aug 19;41(1):147–57. doi: 10.1007/s10067-021-05877-3 (PMC8724104; doi:10.1007/s10067-021-05877-3)
Supplement: Supplementary file 1 — Supplementary file1 (PDF 240 KB) [file 10067_2021_5877_MOESM1_ESM.pdf]

Online Resource. Flow Diagram describing patient enrollment details and evaluation time-points.

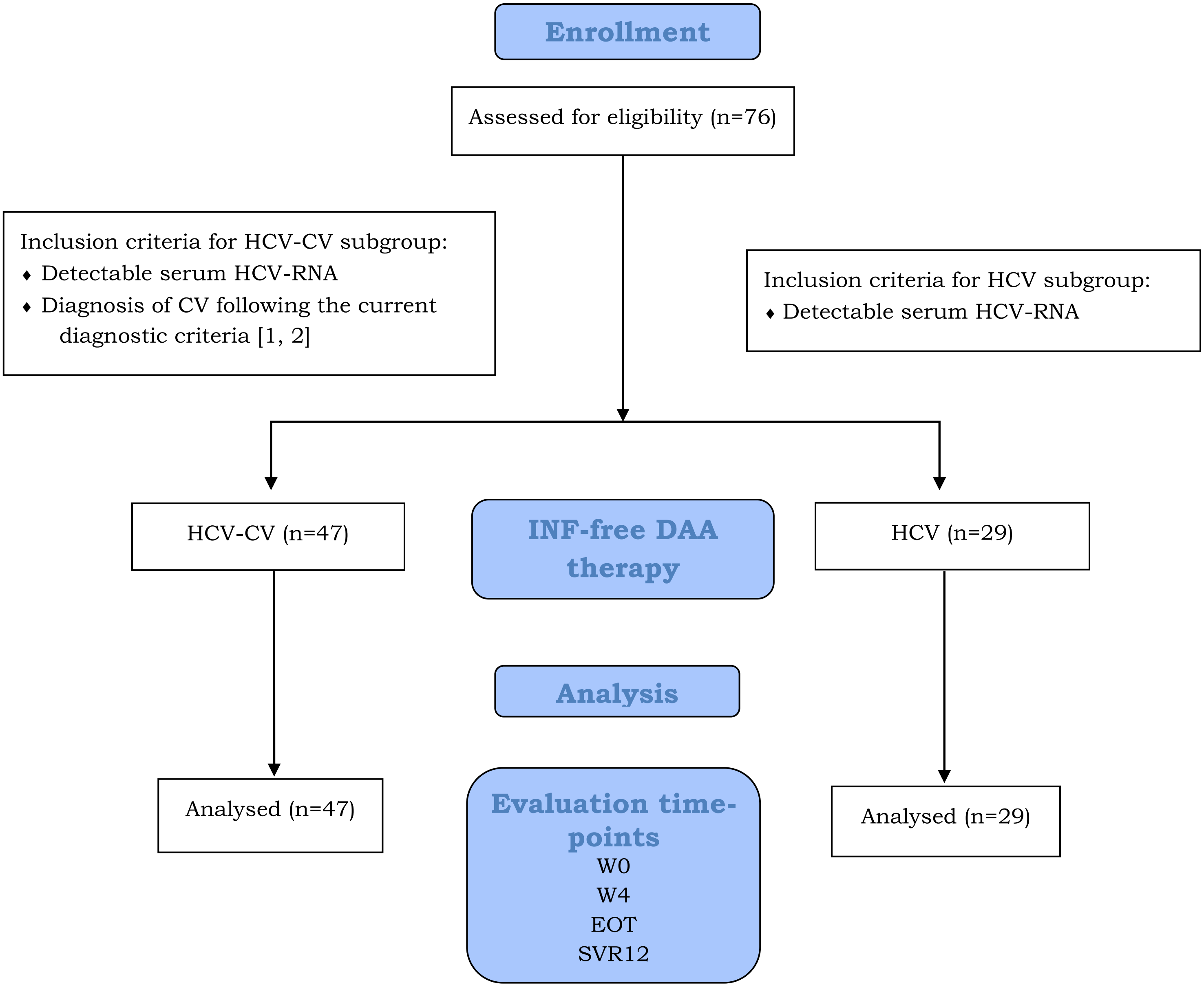

Legend: HCV: Hepatitis C Virus; CV: cryoglobulinemic vasculitis; W0: week 0; W4: week 4; EOT: end of treatment; SVR12: sustained virological response at week 12.

1. De Vita S, Soldano F, Isola M, Monti G, Gabrielli A, Tzioufas A et al. Preliminary classification criteria for the cryoglobulinaemic vasculitis. Ann Rheum Dis 2011;70 7: 1183-90.
2. Quartuccio L, Isola M, Corazza L, Ramos-Casals M, Retamozo S, Ragab GM et al. Validation of the classification criteria for cryoglobulinaemic vasculitis. Rheumatology (Oxford) 2014;53 12: 2209-13.
